# Chilaiditi Syndrome With Colonic Gastrointestinal Stromal Tumor (GIST): An Unusual Overlap

**DOI:** 10.7759/cureus.91900

**Published:** 2025-09-09

**Authors:** Lekhana Dayanand, Krishna Sisir Darbha, Jyothi Halepalya Tukaram, Sathya Lakshmi Mekkoth, Abir Mandal

**Affiliations:** 1 Department of General Practice, Fortis Hospital, Rajajinagar, Bengaluru, IND

**Keywords:** cd117, chilaiditi syndrome, colonic gist, colonic interposition, dog1, gastrointestinal stromal tumor, imatinib, right upper quadrant pain, spindle cell tumor, surgical resection

## Abstract

Chilaiditi syndrome is a rare condition characterized by the interposition of the colon between the liver and diaphragm, often mimicking other abdominal pathologies. While gastrointestinal stromal tumors (GISTs) are the most common mesenchymal tumors of the GI tract, they rarely originate from the colon. We report a 69-year-old man presenting with progressive right upper quadrant pain, low-grade fever, weight loss, altered bowel habits, sluggish bowel sounds, and a positive Murphy's sign. Initial imaging with an erect X-ray showed air under the right hemidiaphragm, prompting suspicion of intra-abdominal pathology. Contrast-enhanced computed tomography (CECT) revealed an interposed ascending colon with a heterogeneously enhancing, necrotic mass and air-fluid level, suggestive of GIST. Positron-emission tomography-computed tomography confirmed a hypermetabolic colonic mass with retroperitoneal and pelvic nodal metastases. Colonoscopy identified a friable, ulcerated lesion, and biopsy confirmed a spindle cell GIST, positive for Cluster of Differentiation 117 and Discovered on GIST-1. Surgical resection was done, after which the patient had an uncomplicated recovery and was initiated on adjuvant imatinib therapy. To the best of our knowledge, this is the first reported case of GIST arising from an interposed colonic segment without prior surgery, emphasizing the need for thorough evaluation in rare presentations.

## Introduction

Chilaiditi syndrome is a rare clinical condition characterized by the interposition of the colon between the liver and the hemidiaphragm [1], with symptoms often mimicking more acute abdominal pathologies [2]. Although it can be asymptomatic, symptomatic presentations can include abdominal pain, distension, nausea, vomiting, altered bowel habits, and, less commonly, arrhythmias, respiratory distress, or chest pain [3]. Gastrointestinal stromal tumors (GISTs), on the other hand, are the most common mesenchymal tumors of the gastrointestinal (GI) tract, typically arising from the stomach or small intestine, with involvement of the colon being rare [4,5]. Approximately 80% of GISTs harbor activating mutations in the receptor tyrosine kinase proto-oncogene, while around 10% exhibit mutations in the platelet-derived growth factor receptor alpha gene [6].

The coexistence of Chilaiditi syndrome and a colonic GIST is exceptionally rare. To the best of our knowledge, there have been no previously reported cases in the literature describing the simultaneous occurrence of Chilaiditi syndrome with a GIST originating specifically from the interposed segment of the colon in the absence of any previous surgical history.

This unique presentation introduces a new diagnostic challenge associated with both conditions and underscores the importance of maintaining a broad differential diagnosis in atypical abdominal presentations. Our case not only contributes a novel finding to existing literature but also highlights the clinical, radiological, and pathological features that guided diagnosis and appropriate management.

## Case presentation

A 69-year-old male patient presented to the outpatient department with complaints of persistent right upper quadrant abdominal pain that had gradually progressed over the past week. The pain was described as dull, nonradiating, and intermittently crampy, with no aggravating or relieving factors. It was associated with low-grade, intermittent fever, unintentional weight loss of approximately 8 kg, and episodes of loose stools occurring two to three times daily over the past month. He denied any history of vomiting, hematochezia, or melena. There was no significant past medical, family, or surgical history, and he was not on any long-term medications.

On physical examination, the patient was hemodynamically stable, with a body mass index of 23.2 kg/m². Abdominal examination revealed tenderness in the right upper quadrant, with positive Murphy's sign, without any signs of guarding or rebound tenderness. The patient also gave a history telling that he was not able to pass flatus and felt bloated for one day. There was no palpable mass or organomegaly, and bowel sounds were sluggish. After admission, the patient passed flatus.

**Figure 1:**
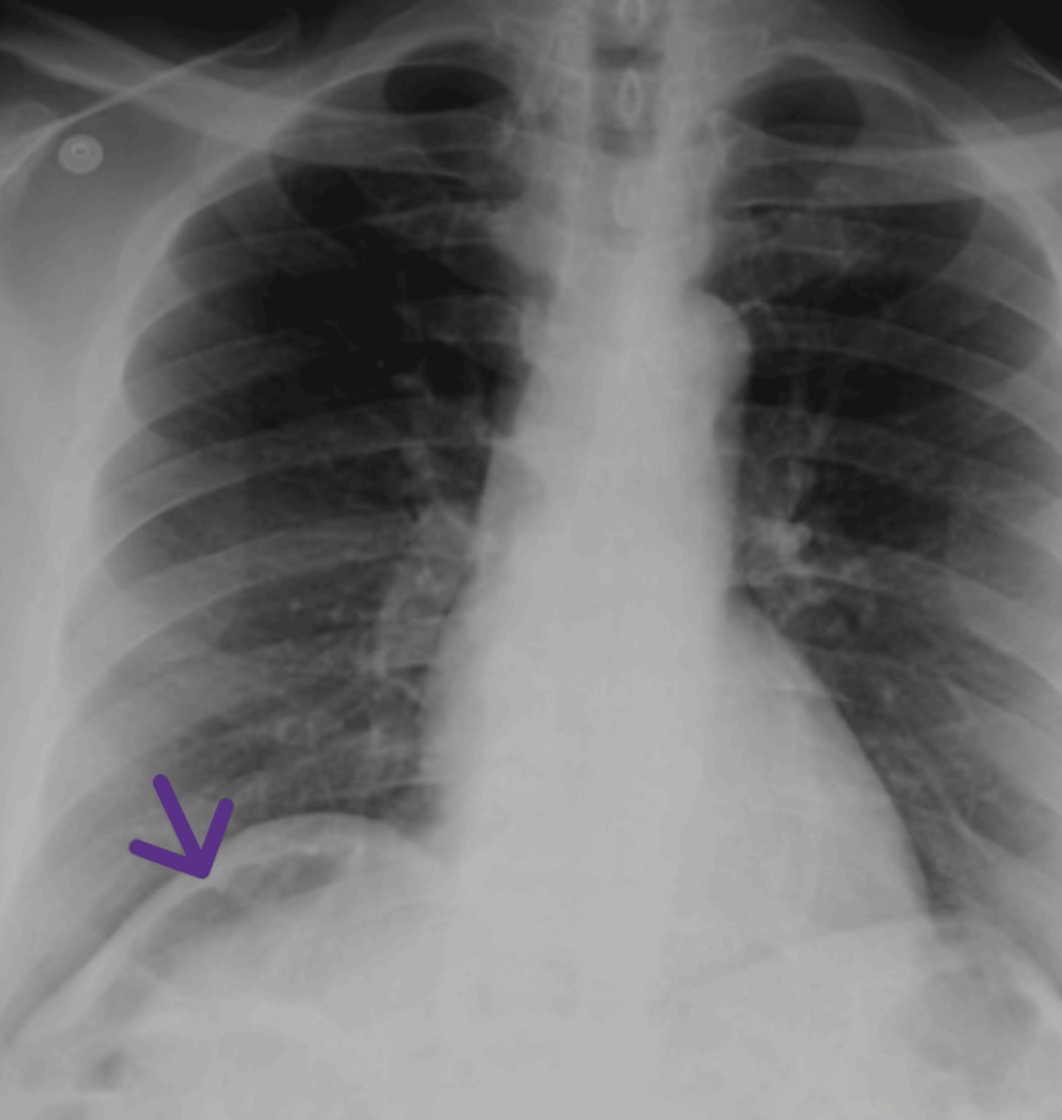
Erect X-ray denoting Chilaiditi sign (pseudopneumoperitoneum) mimicking apparent free intraperitoneal air under the right hemidiaphragm (purple arrow)

Given the clinical presentation, an erect X-ray was performed as an initial investigation. The radiograph demonstrated air under the right hemidiaphragm. The presence of gas overlying the hepatic shadow raised concern for an intra-abdominal pathological process, as shown in Figure 1.

To further evaluate the cause and localize the pathology, a contrast-enhanced computed tomography (CECT) scan of the abdomen was performed. Subsequently, other lab investigations were also ordered. The complete blood count indicated mild microcytic hypochromic anemia. Iron studies were consistent with iron deficiency anemia (IDA), as detailed in Tables 1, 2.

**Table 1:** Complete blood count report

Test name	Value	Normal range
Hemoglobin	11.2 g/dL	Male: 13.5-17.5 g/dL; female: 12-15.5 g/dL
Hematocrit	34.5%	Male: 41-53%; female: 36%-46%
Red blood cell count	4.2 million/μL	Male: 4.7-6.1 million/μL; female: 4.2-5.4 million/μL
White blood cell count	8,000/μL	4,000-11,000/μL
Platelet count	280,000/μL	150,000-450,000/μL
Mean corpuscular volume	79 fL	80-100 fL
Mean corpuscular hemoglobin	25 pg	27-33 pg
Mean corpuscular hemoglobin concentration	33 g/dL	32-36 g/dL
Red cell distribution width	14.2%	11.5%-14.5%
Neutrophils (absolute)	5,000/μL	2,000-7,000/μL
Lymphocytes (absolute)	2,200/μL	1,000-4,800/μL
Monocytes (absolute)	400/μL	200-1,000/μL
Eosinophils (absolute)	150/μL	0-500/μL
Basophils (absolute)	30/μL	0-100/μL

**Table 2:** Iron study blood report

Parameter	Result	Reference range
Hemoglobin	10.8 g/dL	Female: 12-16 g/dL; Male: 13.5-17.5 g/dL
Hematocrit	33%	36%-46%
Mean corpuscular volume	76 fL	80-100 fL
Serum iron	40 µg/dL	60-170 µg/dL
Total iron binding capacity	410 µg/dL	250-400 µg/dL
Transferrin saturation	10%	20%-50%
Serum ferritin	12 ng/mL	Female: 20-200 ng/mL; male: 30-300 ng/mL
Reticulocyte count	0.8%	0.5%-2.0%

Stool routine examination was positive for occult blood, suggestive of GI blood loss. No visible blood or parasitic elements were observed, though rare red blood cells were noted microscopically. Inflammatory markers, including lactate dehydrogenase (LDH), C-reactive protein (CRP), and erythrocyte sedimentation rate (ESR), were elevated, pointing toward a systemic inflammatory response. Renal and liver function tests were within normal limits, and serum electrolytes were unremarkable, as detailed in Tables 3-6, respectively.

**Table 3:** Stool routine examination report HPF: high-power field

Parameter	Result	Reference range
Color	Brown	Brown
Consistency	Semiformed	Formed/semiformed
Mucus	Absent	Absent
Blood (visible)	Absent	Absent
Occult blood	Positive	Negative
Pus cells	0-2/HPF	0-5/HPF
Red blood cells	Rare	Absent/rare
Ova/cysts/parasites	Not seen	Not seen
Fat globules	Not seen	Not seen
Undigested food particles	Few	Few
Reaction (pH)	Slightly acidic	Neutral to slightly acidic

**Table 4:** Inflammatory markers

Parameter	Result	Reference range
Lactate dehydrogenase	260 U/L	100-190 U/L
C-reactive protein	18 mg/L	<5 mg/L
Erythrocyte sedimentation rate	48 mm/hour	Male: 0-20 mm/hour; female: 0-30 mm/hour

**Table 5:** Liver function test

Test	Value	Normal range
Alanine aminotransferase	25 U/L	7-56 U/L
Aspartate aminotransferase	24 U/L	10-40 U/L
Alkaline phosphatase	89 U/L	40-129 U/L
Total bilirubin	0.7 mg/dL	0.2-1.2 mg/dL
Direct (conjugated) bilirubin	0.2 mg/dL	0.1-0.3 mg/dL
Indirect (unconjugated) bilirubin	0.6 mg/dL	0.2-0.9 mg/dL
Gamma-glutamyl transferase	30 U/L	Men: 9-48 U/L; women: 8-35 U/L
Total protein	7 g/dL	6.3-7.9 g/dL
Albumin	4.2 g/dL	3.5-5.0 g/dL
Globulin	3 g/dL	2.0-3.5 g/dL
Albumin/globulin ratio	1.4	1.0-2.5

**Table 6:** Renal function test with serum electrolytes BUN: blood urea nitrogen; GFR: glomerular filtration rate

Tests	Patient value	Normal range
Blood urea nitrogen	15 mg/dL	7-20 mg/dL
Serum creatinine	0.9 mg/dL	Male: 0.7-1.3 mg/dL; female: 0.6-1.1 mg/dL
BUN/creatinine ratio	15:1	10:1-20:1
Serum uric acid	5.2 mg/dL	Male: 3.5-7.2 mg/dL; female: 2.6-6 mg/dL
Estimated GFR	105 mL/minute/1.73 m²	>90 mL/minute/1.73 m²
Serum electrolytes		
Sodium	139 mEq/L	135-145 mEq/L
Potassium	4.2 mEq/L	3.5-5.0 mEq/L
Chloride	102 mEq/L	98-107 mEq/L
Bicarbonate	24 mEq/L	22-28 mEq/L

**Figure 2:**
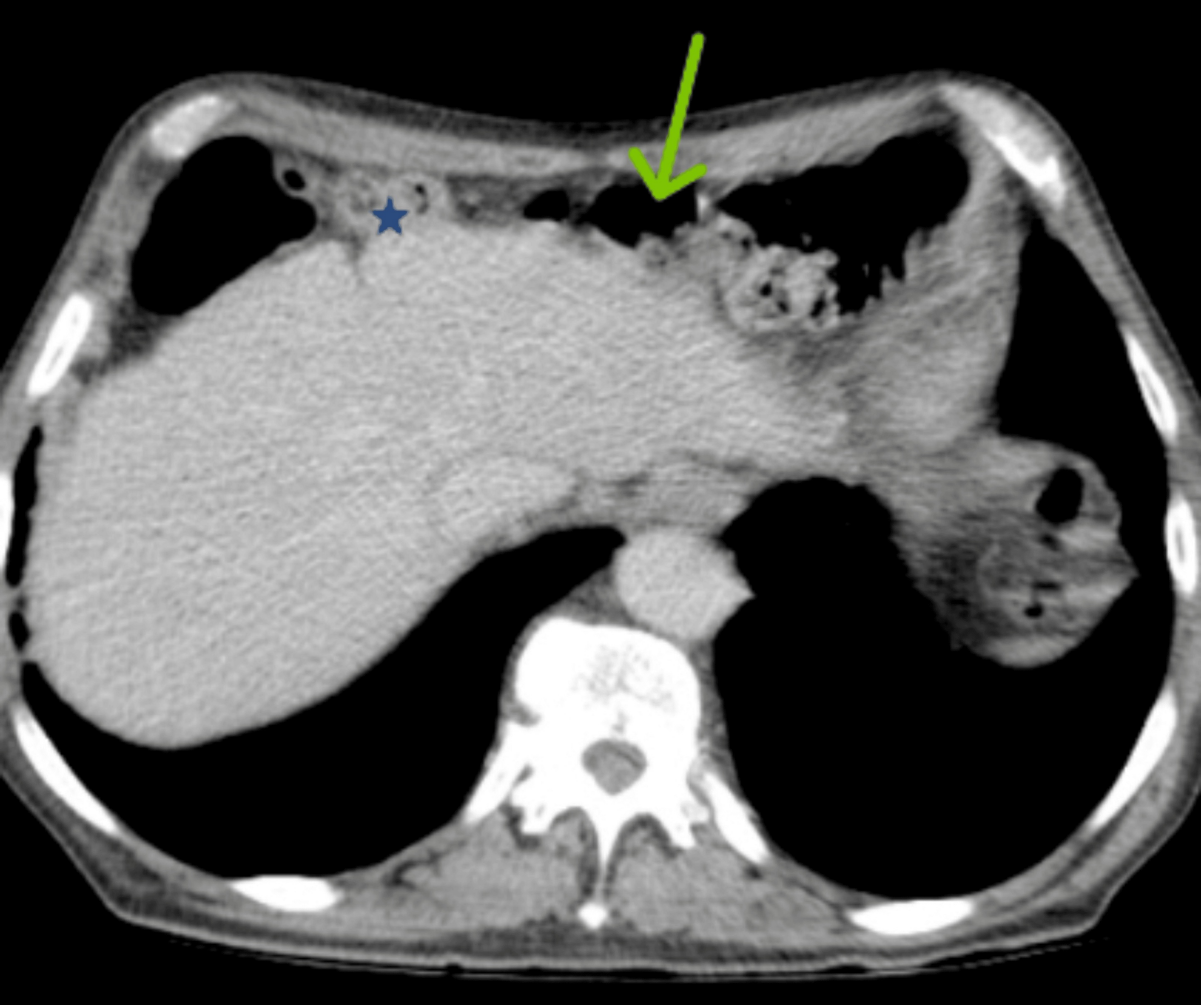
CECT image showing interposition of the ascending colon between the liver and the right hemidiaphragm, as denoted by the green arrow, a well-defined, heterogeneous mass lesion arising from the interpositioned ascending colon, characterized by central areas of necrosis and the presence of an air-fluid level within the lesion, as denoted by the blue star, consistent with the Torricelli-Bernoulli sign CECT: contrast-enhanced computed tomography

**Figure 3:**
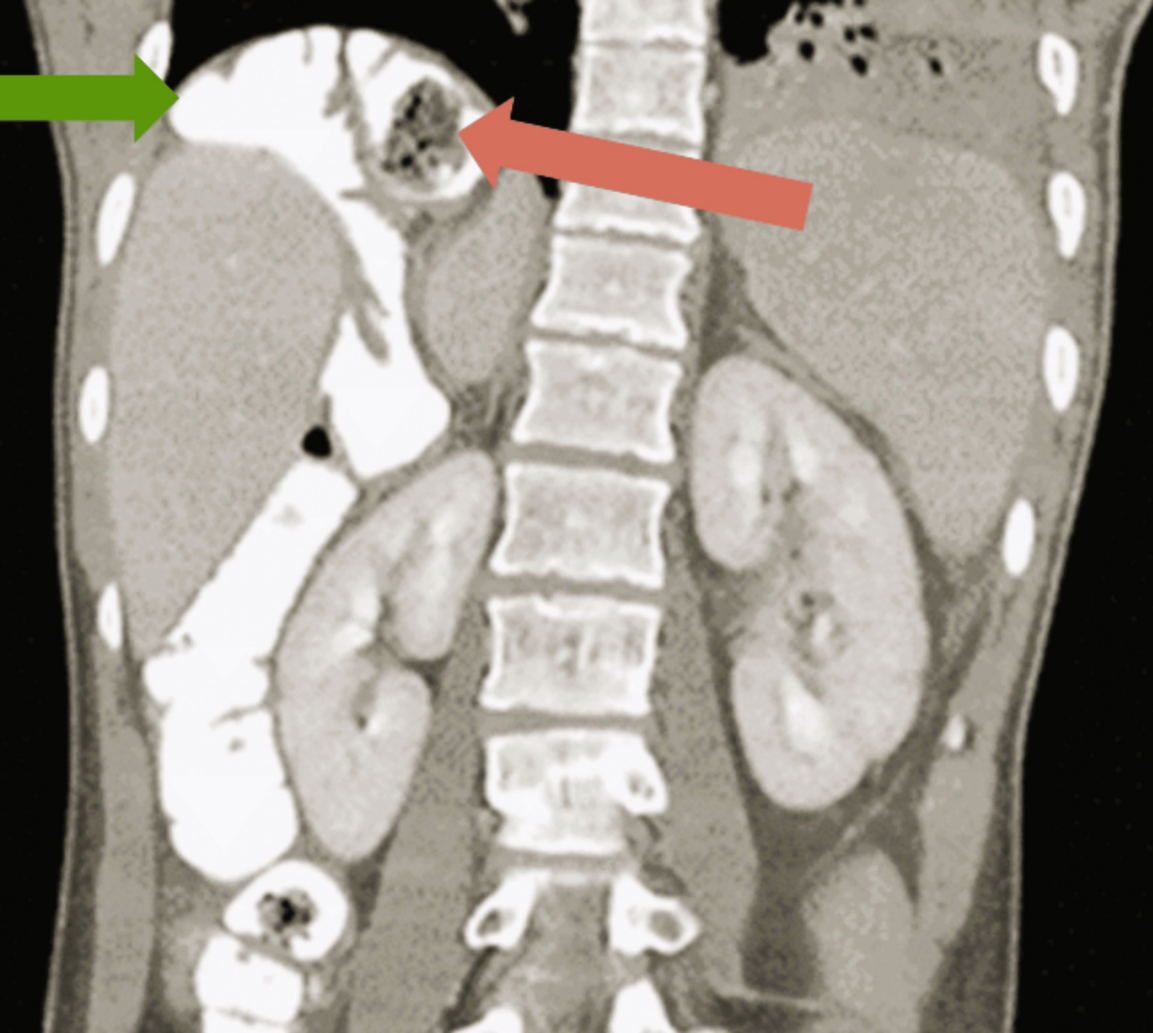
Coronal CECT image showing ascending colon between the liver and the right hemidiaphragm, as denoted by the green arrow, and the lesion, as pointed by the orange arrow CECT: contrast-enhanced computed tomography

CECT imaging revealed normal hepatobiliary anatomy with interposition of the ascending colon between the liver and the right hemidiaphragm, a well-defined, heterogeneous mass lesion arising from the interpositioned ascending colon, characterized by central areas of necrosis and the presence of an air-fluid level within the lesion. This finding was consistent with the Torricelli-Bernoulli sign, which typically indicates communication of the tumor with the GI lumen or internal cavitation, a feature sometimes seen in GIST, as seen in Figures 2, 3.

**Figure 4:**
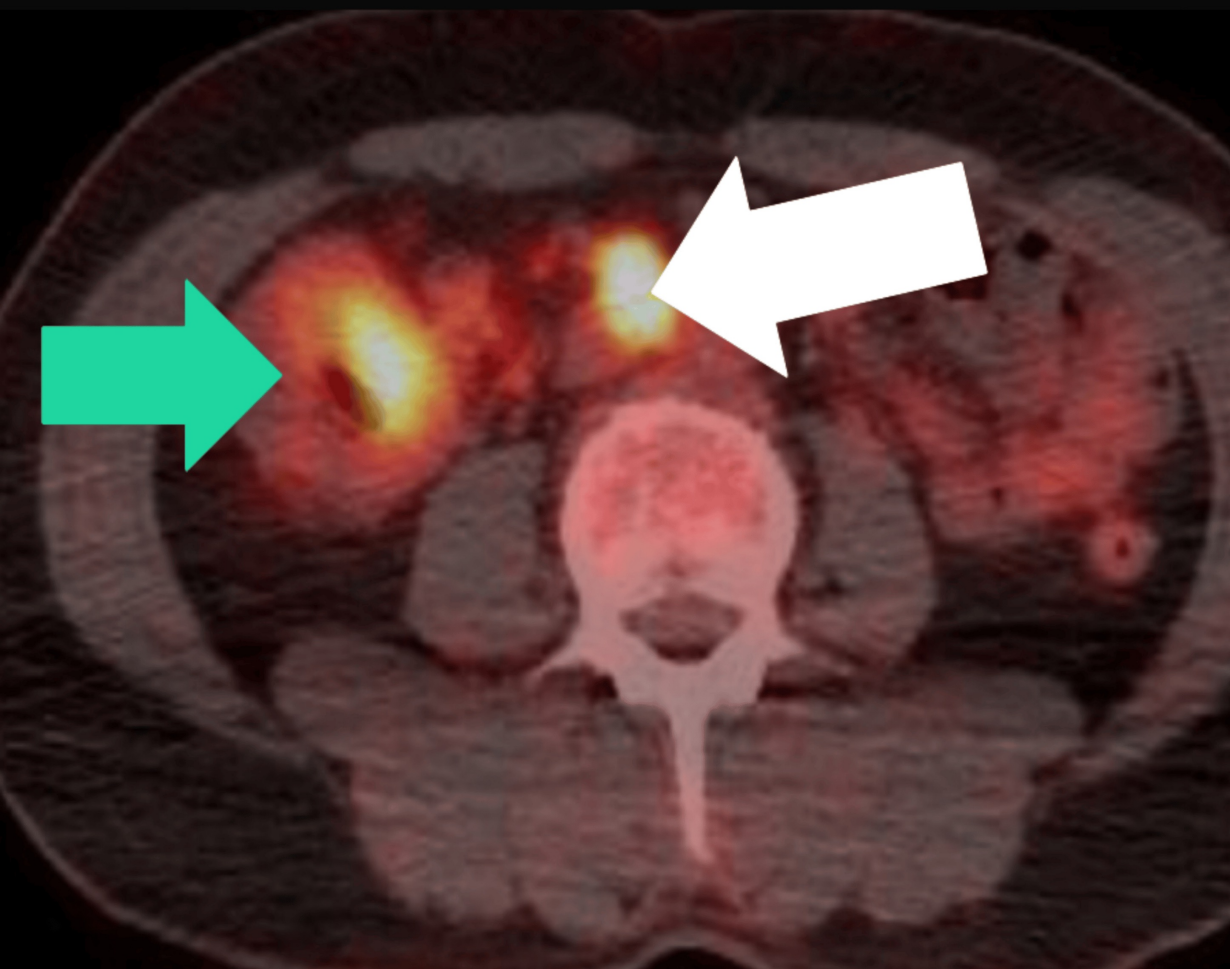
PET-CT image showing a hypermetabolic mass lesion localized to the ascending colon, as pointed by the green arrow, with evidence of distant metastasis with hypermetabolic lymph nodes, as pointed by the white arrow PET-CT: positron-emission tomography-computed tomography

In light of these findings, a whole-body positron-emission tomography-computed tomography (PET-CT) scan was conducted to assess the metabolic activity and potential spread of the lesion, which demonstrated a hypermetabolic mass lesion localized to the ascending colon, with evidence of distant metastasis with hypermetabolic retroperitoneal and pelvic lymph nodes, as seen in Figure 4.

**Figure 5:**
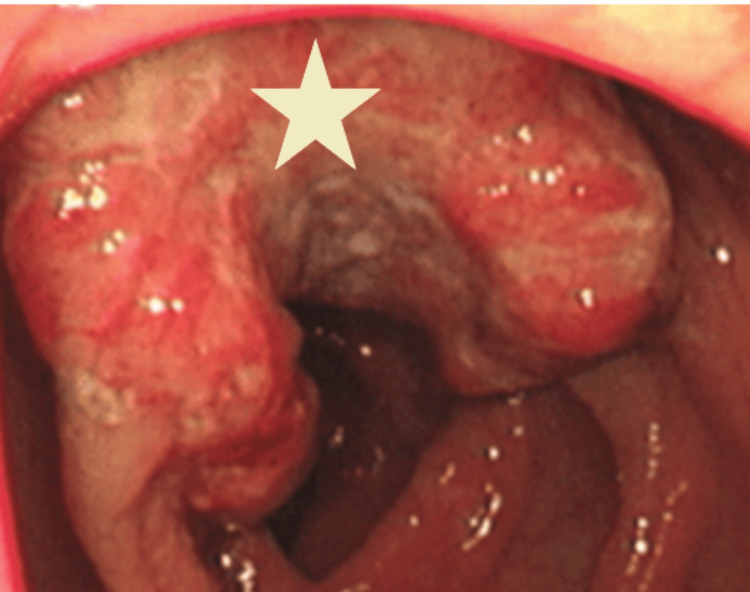
A friable, ulcerated intraluminal mass in the ascending colon seen on colonoscopy, denoted by the star

To obtain tissue diagnosis, the patient underwent colonoscopy, which revealed a friable, ulcerated intraluminal mass in the ascending colon, as seen in Figure 5.

**Figure 6:**
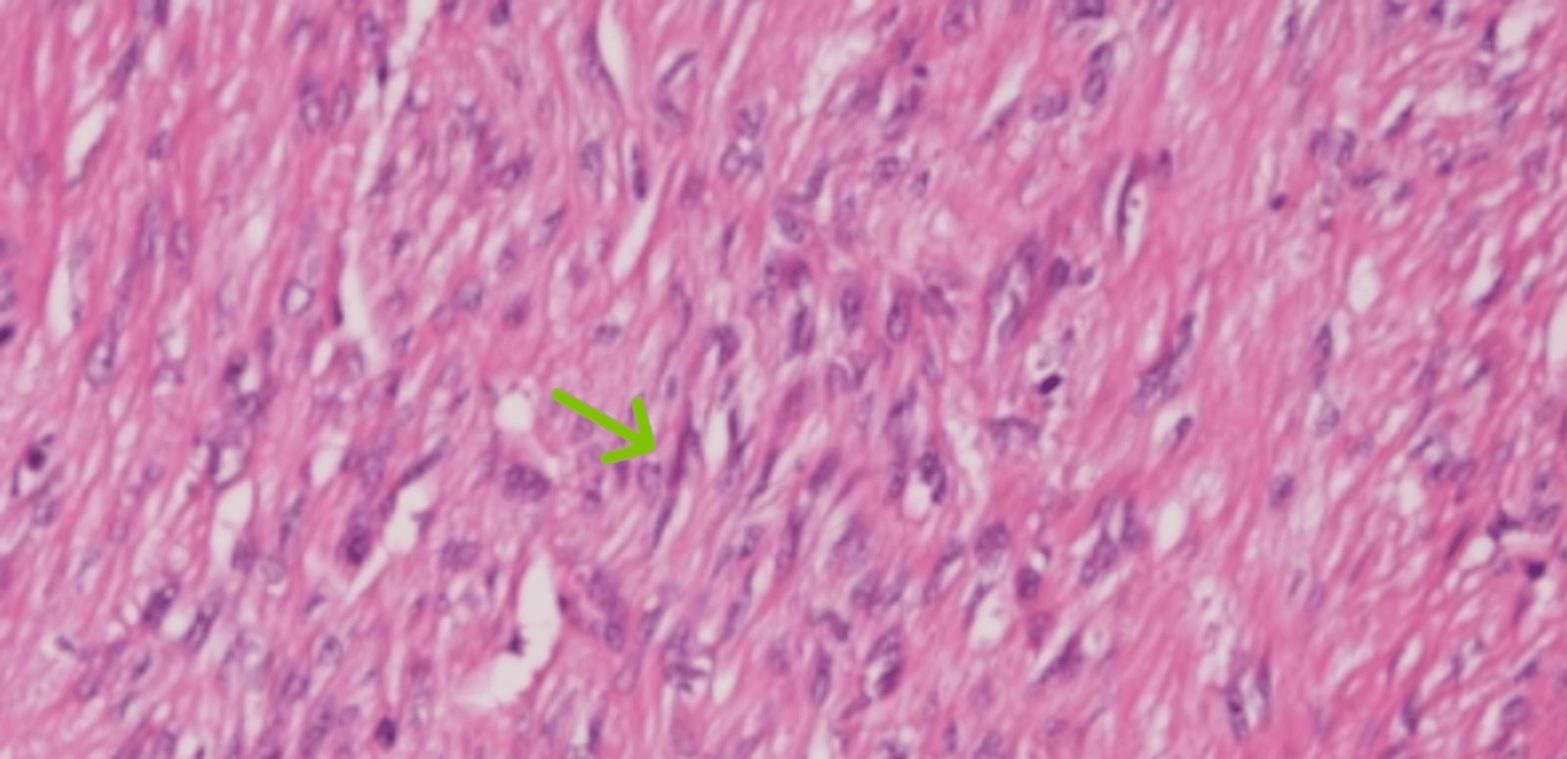
Histopathological examination showing spindle-shaped tumor cells arranged in short fascicles (green arrow), with necrosis, high-grade mitotic activity, and lymphovascular invasion, consistent with the spindle cell subtype of GIST GIST: gastrointestinal stromal tumor

Multiple biopsies were taken from the lesion. Histopathological examination showed spindle-shaped tumor cells arranged in short fascicles, with necrosis, high-grade mitotic activity, and lymphovascular invasion, consistent with the spindle cell subtype of GIST, as seen in Figure 6. Immunohistochemistry revealed positivity for Cluster of Differentiation 117 (CD117) and Discovered on GIST-1 (DOG1), with moderate diffuse positivity for smooth muscle actin (SMA).

Following a multidisciplinary team discussion, surgical intervention was planned. The patient subsequently underwent an exploratory laparotomy, which confirmed the presence of a large colonic mass with retroperitoneal metastasis. A segmental colonic resection with primary anastomosis with lymph node dissection was performed. The resected specimen was sent for histopathological evaluation to confirm the diagnosis and assess margins and mitotic activity.

The patient's postoperative course was uneventful. He was started on early enteral feeding and discharged in stable condition. The patient is currently receiving adjuvant therapy with imatinib.

## Discussion

The Chilaiditi sign, first described by Greek radiologist Demetrius Chilaiditi in 1910, is a rare radiological finding with an incidence of 0.025%-0.28%, characterized by the interposition of the colon between the liver and diaphragm, which occurs four times more often in men than in women [1]. It arises from anatomical or functional abnormalities that allow colon interposition. Causes include ligament laxity, dolichocolon, chronic constipation, lung disorders, cirrhosis, obesity, ascites, and neuropsychiatric disorders, all of which alter the intra-abdominal anatomy or pressure [2].

The Chilaiditi sign is asymptomatic, whereas Chilaiditi syndrome refers to symptomatic bowel interposition. It can present with abdominal discomfort, nausea, bloating, altered bowel habits, and, less commonly, arrhythmias, chest pain, or respiratory distress [3]. Chilaiditi sign is diagnosed by: 1) bowel interposed between the liver and elevated right hemidiaphragm, 2) air-filled colon mimicking pneumoperitoneum, and 3) liver displaced below the level of the left hemidiaphragm. Management ranges from conservative therapy to surgery. Due to its rarity, especially in India, it is frequently misdiagnosed or is rarely considered as a differential diagnosis in clinical settings.

GISTs, on the other hand, are the most common mesenchymal tumors of the GI tract, typically arising from the stomach or small intestine, with colonic origin being notably rare [4,5]. Colonic GISTs represent a rare subset, accounting for only 1%-2% of all GISTs and approximately 0.1% of all large intestinal tumors [6].

To our knowledge, this is the first reported case of a GIST originating specifically from an interposed segment of colon in a patient with Chilaiditi syndrome and no prior abdominal surgery, an unusual convergence of two rare entities.

Previous reports of GIST arising in colonic interpositions have been documented, such as the case by Badshah et al., where a GIST developed in an esophageal resection and colonic pull-through performed after caustic injury [7]. However, unlike this, our patient had no antecedent surgical history, underscoring the uniqueness of our report and expanding the differential diagnosis of both colonic GISTs and Chilaiditi syndrome.

Our patient's presentation with right upper quadrant pain, low-grade fever, weight loss, and altered bowel habits initially suggested a hepatobiliary or hollow viscus pathology. The presence of subdiaphragmatic gas on an erect X-ray mimicked pneumoperitoneum, a frequent diagnostic pitfall in Chilaiditi syndrome. However, CECT revealed colonic interposition with a heterogeneously enhancing mass and air-fluid level consistent with the Torricelli-Bernoulli sign, which typically indicates either tumor necrosis or luminal communication, features sometimes seen in aggressive GIST [8].

Subsequent PET-CT confirmed a hypermetabolic lesion with retroperitoneal and pelvic nodal metastases. Colonoscopy and biopsy revealed a friable, ulcerated intraluminal mass, and histopathology confirmed a spindle cell subtype of GIST, characterized by diffuse positivity for CD117 and DOG1, which are hallmark immunohistochemical markers for GISTs. SMA positivity, also noted, is seen in a subset of GISTs [9,10].

While GISTs typically metastasize hematogenously to the liver or peritoneum, lymphatic spread is relatively rare. However, the presence of lymphadenopathy in this case, combined with a high mitotic index, extensive necrosis, and lymphovascular invasion, reflects an aggressive biological behavior [11].

IDA is commonly seen in GISTs due to chronic mucosal bleeding caused by the overlying epithelial ulceration. Miettinen and Lasota emphasize that mucosal ulceration is common in GISTs, especially in nongastric locations, and often results in IDA [5]. A positive stool occult blood test is an early indicator of GISTs. Joensuu highlights that occult GI bleeding is a frequent manifestation in GISTs and should prompt further evaluation for submucosal lesions like GISTs [11]. Elevated CRP, LDH, and ESR levels are often associated with colonic tumors, as seen in our case [12,13].

Surgical resection remains the cornerstone of treatment for localized or resectable GISTs. Our patient underwent successful segmental colonic resection and anastomosis with lymphadenectomy, followed by adjuvant Imatinib, which is indicated in high-risk or metastatic GISTs to reduce recurrence and improve survival [14]. The patient’s postoperative course was uneventful, and he was discharged on imatinib therapy with plans for regular oncological follow-up.

This case highlights several key clinical lessons, including the importance of recognizing Chilaiditi syndrome as a potential masquerader of more acute abdominal conditions, the diagnostic value of advanced imaging in atypical presentations, and the necessity of considering rare pathologies, such as colonic GISTs, in patients with nonspecific symptoms. Additionally, it reinforces the value of multidisciplinary evaluation in managing complex cases involving anatomical variants and rare malignancies.

## Conclusions

Our case highlights a unique convergence of two distinct pathologies, Chilaiditi syndrome, and colonic GIST, in a patient with no prior surgical history. The presentation, initially suggestive of hepatobiliary pathology, and the detection of subdiaphragmatic gas, initially mimicking pneumoperitoneum, highlight the diagnostic challenge posed by such clinical scenarios. The combination of advanced imaging and histopathological examination played a pivotal role in reaching the diagnosis. This report emphasizes the importance of maintaining a broad differential diagnosis when confronted with nonspecific abdominal symptoms. It also illustrates the value of multidisciplinary involvement to eliminate delay in getting to the diagnosis and to save the best possible outcome when the seemingly familiar clinical presentation takes an unexpected twist. Surgical resection followed by adjuvant Imatinib therapy remains the cornerstone for managing high-risk or metastatic GISTs. To our knowledge, this is the first reported case of a colonic GIST arising from an interposed segment in the context of Chilaiditi syndrome without prior surgical intervention, thereby contributing novel insights to the existing literature.
